# Anemia Is a Risk Factor for the Development of Ischemic Stroke and Post-Stroke Mortality

**DOI:** 10.3390/jcm10122556

**Published:** 2021-06-09

**Authors:** Jayoon Heo, Tae-Mi Youk, Kwon-Duk Seo

**Affiliations:** 1 Department of Hematology-Oncology, National Health Insurance Service Ilsan Hospital, 100 Ilsan-ro, Ilsan-donggu, Goyang 10444, Korea; jayoonheo@nhimc.or.kr; 2 Research Institute, National Health Insurance Service Ilsan Hospital, 100 Ilsan-ro, Ilsan-donggu, Goyang 10444, Korea; tmyouk@nhimc.or.kr; 3 Department of Statistics, Korea University, 145 Anam-ro, Seongbuk-gu, Seoul 02841, Korea; 4 Department of Neurology, National Health Insurance Service Ilsan Hospital, 100 Ilsan-ro, Ilsan-donggu, Goyang 10444, Korea

**Keywords:** anemia, ischemic stroke, mortality

## Abstract

Background: anemia is known to be a risk factor for developing ischemic stroke in long-term follow-up studies, and it is also known to increase the risk of death in ischemic stroke patients. We aimed to determine the association of anemia with the risk of ischemic stroke and the risk of death after ischemic stroke. Methods: The study included patients from National Health Insurance Service cohort, from January 2005 to December 2015. Anemia patients were defined as those with confirmed diagnostic codes and related medications in the sample cohort, and patients under the age of 18 were excluded. To perform a comparative analysis with the control group, twice as many patients were extracted by propensity score matching. The effects of anemia on the development of ischemic stroke were analyzed. Results: A total of 58,699 patients were newly diagnosed with anemia during the study period. In anemia group, the rate of ischemic stroke occurring within 1 year was 0.550%, and the rate was 0.272% in the control group. The odds ratio of anemia related to ischemic stroke was 1.602 (95% confidence intervals (CI) 1.363–1.883). During the follow-up period, 175 out of 309 (56.6%) died in anemia group, and 130 out of 314 (41.4%) died in control group. The anemia group showed a higher risk of death than the control group (Hazard ratio 1.509, 95% CI 1.197–1.902). Conclusion: Analysis of the nationwide health insurance data revealed that anemia is one of the risk factors for the development of ischemic stroke, and also an independent prognostic factor affecting post-stroke mortality.

## 1. Introduction

Anemia is a decrease in red blood cells (RBCs) or hemoglobin in the blood. The most common cause of anemia is a blood loss, and insufficient RBC production or increased breakdown of RBCs can be causes as well. Anemia is a common disease in about 10% of people aged ≥65 years and its prevalence increase with age [1]. Ischemic stroke is frequently accompanied by anemia; previous studies have confirmed an average prevalence rate of 15–20% [2,3,4,5,6], and a maximum prevalence of 30% [7]. These prevalence rates of anemia are not significantly different from the 33% for diabetes or atrial fibrillation, which are known major risk factors for developing ischemic stroke [8,9]. In a previous long-term observational study, anemia in women increased the risk of ischemic stroke [10]. In subsequent observational studies, high or low hemoglobin levels increased the developmental risk of ischemic stroke for both men and women [11]. In patients with chronic kidney disease accompanied by anemia, the reported risk for stroke is high [12]. In patients with ischemic stroke, a history of iron-deficiency anemia was more prevalent than in those without [13].

Anemia alone is known to increase mortality risks in older adults [1], and when accompanied by cardiac diseases [14,15]. An observational study has shown that anemia at the time of hospitalization in patients with ischemic stroke increases the risk of death upon long-term follow-up [16]. In a meta-analysis of observational studies lasting >1 year, mortality risk for both ischemic stroke and hemorrhagic stroke increased when accompanied by anemia [4,17]. Additionally, a recent study confirmed that patients who were anemic at the time of an ischemic stroke had a high risk for recurrence and development of cardiovascular disease and cardiovascular death within a year [2]. Furthermore, it was reported that delayed treatment of anemia during hospitalization with ischemic stroke was associated with poor outcome at 3 months [6]. Therefore, anemia is a risk factor for developing ischemic stroke and increases the chance of recurrence and mortality risk. While there have been many cohort studies, no study has investigated data representing one nation’s entire population. We analyzed health insurance claims data to confirm the development risk of ischemic stroke due to anemia; we followed up with patients who developed ischemic stroke to analyze whether anemia influenced the post-stroke mortality risk.

## 2. Materials and Methods

### 2.1. Study Population

The National Health Insurance Service (NHIS) in South Korea is medical insurance to which 97% of all South Koreans have registered. The NHIS manages the claims data of all medical practices covered by insurance. This study used the national sample cohort data from the NHIS, created with about a million samples representing about 2% of the entire population, extracted out of all NHI claims data using a stratified random method with sex, age, address and insurance cost as references. This sample cohort is a validated dataset that represents the population in terms of the prevalence of major diseases [18]. Our study was conducted for the standard cohort in 1 January 2005–31 December 2015.

Data from 1 January 2005 to 31 December 2005, and 1 January 2015 to 31 December 2015 were excluded to take washout and follow-up periods into account; thus, study subjects were extracted from the nine years of data for 2006–2014. Subjects <18 years old were excluded because our focus was on adult patients. Diagnoses in the sample cohort data were categorized based on the International Classification of Diseases, 10th Revision codes (ICD-10).

Patients were selected for our study if they had claims that occurred for primary diagnoses of: D50. Iron deficiency anemia; D51. Vitamin B12-deficiency anemia; D52. Folate deficiency anemia; D53. Other nutritional anemia; D55. Anemia due to enzyme disorders; D59. Acquired hemolytic anemia; D61. Other aplastic anemia; D63. Anemia in chronic diseases classified elsewhere; D64. Other anemias. The hemoglobin level was unavailable in the cohort data, so the following method was used to improve the analysis accuracy regarding patients diagnosed with anemia. For those hospitalized, the hemoglobin level was identified from tests performed on admission, so any of the above diagnoses in admission claims data were selected as subjects. In out-patient treatment claims data, the above diagnostic codes can be claimed to not contain hemoglobin levels, so subjects had treatment medication prescribed. We extracted data if patients diagnosed with D50, D53, D63 and D64 had medications prescribed for treating iron-deficiency anemia; if those diagnosed with D51, D52, D53, D61, D63 and D64 had medications prescribed for treating aplastic anemia (cobalamin, folic acid); if those diagnosed with D63 were prescribed erythropoietin. Out of the billing statements satisfying the above conditions, the start date of medical treatment was defined as the time of confirmation of anemia; with this time point as a reference, the development of ischemic stroke or related death was searched. If the diagnosis code appears multiple times within a person, only the first claim was confirmed as newly diagnosed with anemia. Patients who received a claim with an anemia diagnosis but did not receive a relevant prescription, and patients who only claimed an anemia treatment drug without a diagnosis were not included as study subjects. In total, 59,905 patients received their first diagnosis in 2006–2014. The diagnosed anemic patients were compared with those not in terms of developing ischemic stroke and death; a control group was created based on 1:2 propensity score matching (PSM) with sex and age (years) as references. The remaining 58,699 patients were used for analysis in pairs.

The developmental risk of ischemic stroke is high when accompanied by diseases such as hypertension, diabetes, dyslipidemia, atrial fibrillation and chronic kidney disease. To evaluate the influence of accompanying anemia on the development of ischemic stroke, we confirmed the presence or absence of accompanying disease in previous claims data from the time of anemia diagnosis. In the control group, it was confirmed using the paired anemia patient’s diagnosis date as the index date. It was not possible to extract the propensity score matching, since the matching with the control group should be based on the same time with the newly diagnosed anemia. Therefore, it was not possible to extract so that the proportions of comorbid diseases were the same in the anemia group and the control group. Out of 817,645 subjects who had never been diagnosed with anemia gathered during the study period, 117,398 subjects—double the number of study subjects—were extracted after PSM with sex and age at the time of identifying the study subject as references. They were combined with the diagnosed anemic patients and a total of 176,097 were determined as subjects for our analysis. In order to accurately assess the risk of anemia on the development of ischemic stroke, patients with a history of ischemic stroke were excluded from the risk analysis. 2510 patients in the anemia group and 1763 patients in the control group had a history of ischemic stroke, so they were excluded from the risk analysis. This study was approved by the Institutional Review Board of the National Health Insurance Service Ilsan Hospital (NHIMC 2020-03-035).

### 2.2. Outcome

Ischemic stroke development before and after the diagnosis of anemia was identified, and the number of deaths including all causes of mortality were confirmed during the follow-up observational period. The development of ischemic stroke was defined as admission claims under “I63. Ischemic stroke” in ICD-10 codes. To improve the accuracy of analysis, the admission claims had to meet all of the following three criteria. The number of claimed hospitalization was ≥3 days, a billing code exists for brain magnetic resonance imaging (MRI) testing, and billing codes exist for treatment such as antiplatelet drugs, anticoagulants, intravenous thrombolytic drugs and intra-arterial thrombectomy. The accuracy of diagnoses extracted from claims data this way was verified in a previous study, which reported 84–97% for specificity and 89–95% for positive predictive value [19].

### 2.3. Statistical Analysis

Data analysis was conducted using the statistical software SAS version 9.4 (SAS Institute Inc., Cary, NC, USA); a Chi-square test was performed to compare the frequencies of the accompanying diseases. Multiple logistic regression analysis was performed to identify the accompanying diseases’ risk level on the development of ischemic stroke. Multiple logistic regression analysis was conducted separately for sex and age to identify the risk of anemia in the development of ischemic stroke. Statistical significance was defined as <0.05 in a two-tailed test. Bonferroni correction was conducted to correct errors that may occur in multiple comparisons.

## 3. Results

**Figure 1:**
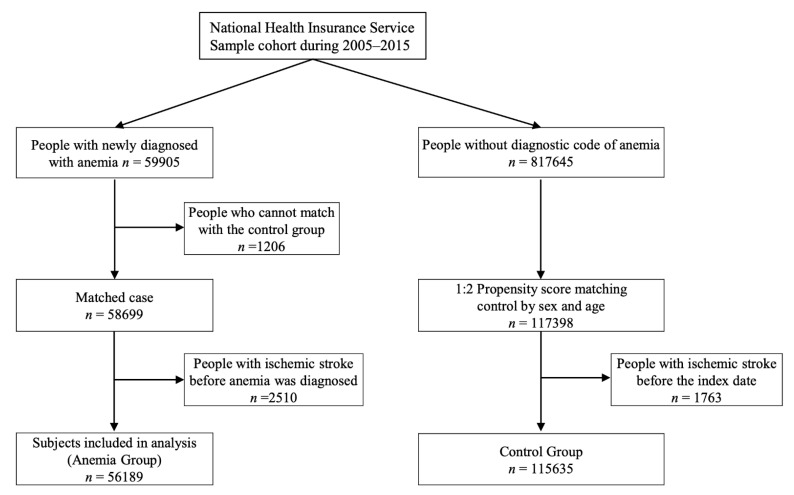
The flowchart of the study patients.

Out of the 58,699 who were ≥18 years old and newly diagnosed with anemia in 2006–2014, 2510 patients were diagnosed with ischemic stroke before the index date (date of diagnosis). Out of 117,398 subjects extracted using PSM, 1174 patients were diagnosed with ischemic stroke before the index date (Figure 1). The proportion of women in anemia group was 67.8%. All the proportion of comorbidities that increase the risk of ischemic stroke were statistically higher in anemia group than the control group (Table 1). The number of patients who developed ischemic stroke within one year after receiving a diagnosis of anemia was 309: incidence rate 0.550%; within two years, 497 and 0.885%. The incidence rates of ischemic stroke in the control group were 0.272% within one year and 0.515% within two years. The number of patients who developed ischemic stroke each year during the follow-up observational period did not differ significantly for the control group. In contrast, the incidence rate of ischemic stroke within one year was higher than in the following year for a new diagnosis of anemia.

Multiple logistic regression analysis was conducted to identify the risk factors for the development of ischemic stroke after a new diagnosis of anemia. The adjusted odds ratio (OR) of anemia related to the development of ischemic stroke within one year was 1.602 (95% confidence intervals (CI): 1.363–1.883); although this was lower than the adjusted OR values for hypertension, diabetes and atrial fibrillation, it was statistically significant (Table 2). Out of the patients diagnosed with anemia, 34,350 were ≥50 years old, comprising 58.5% of the total. The analysis was performed after dividing into two distinct groups in terms of age, i.e., ≥50 years old and <50 years old, regarding the risk factor for ischemic stroke development within two years after the diagnosis of anemia. The adjusted OR value of anemia was 1.414 (95% CI: 1.247–1.605, *p* < 0.0001) for the ≥50 years old group and 2.404 (95% CI: 1.232–4.689, *p* = 0.010) for the <50 years old group. Gender-separated analysis revealed that anemia was still associated with the risk of developing ischemic stroke in women (adjusted OR 1.485 and 95% CI: 1.254–1.759, *p* < 0.0001), but no statistical significance was found in men (adjust OR 1.243, 95% CI 1.037–1.489, *p* = 0.0185) (Table 3).

**Figure 2:**
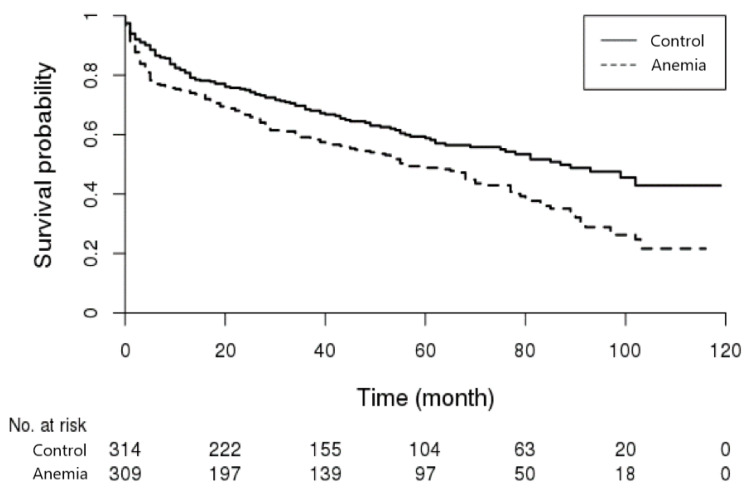
Kaplan-Meier survival curve of the patients with ischemic stroke. Survival rate was lower in patients with anemia.

Out of the patients who developed ischemic stroke within a year after the diagnosis of anemia, those who passed during the follow-up period were 175 out of the 309 in the anemia group (56.6%, mean survival time: 27.9 months), and 130 out of the 314 in the control group (41.4%, mean survival time: 25.4 months). In the Kaplan–Meier survival analysis, the anemia group showed a higher mortality rate (log-rank test *p* = 0.0005) (Figure 2). Cox-regression analysis was performed to investigate the influence of anemia on the mortality risk. Anemia’s hazard ratio (HR) was 1.509 (95%, CI: 1.197–1.902, *p* = 0.0003). Out of all the comorbidities, only anemia and atrial fibrillation were statistically significant, and which was similar for patients ≥50 years old (Table 4). Among patients <50 years old, few passed, so this was not analyzed statistically.

## 4. Discussion

Similar to previous studies’ results, this study identified anemia as a disease that can be a risk factor for ischemic stroke development and is associated with post-stroke mortality risk. In previous long-term follow-up observational studies, existing anemia was shown to increase the risk for ischemic stroke development by about 1.5-fold [11,20]. In this study, the presence of anemia was associated with a higher risk of ischemic stroke development by about 1.6-fold within one year and 1.35-fold within two years, which is similar to previous studies’ findings. The risk for ischemic stroke development was higher within one year after the diagnosis of anemia. The proposed causes for the result are as follows: The risk for ischemic stroke development may have been reduced as the anemic state resolves over time after treatment subject to the diagnosis of anemia because this study selected patients prescribed with drugs to treat anemia as study subjects. The risk for ischemic stroke may decrease if bodily changes occur to adapt to the anemic state over several hours. A previous study has reported that the degree of hemoglobin reduction—rather than the baseline hemoglobin level itself—was associated with ischemic stroke and death after surgery [21]. Other studies have reported a much higher risk of ischemic stroke development in patients who showed a tendency of decreasing hemoglobin level than in those with stable hemoglobin level with no change [22]. Our study subjects were newly diagnosed with anemia, so the hemoglobin level had likely changed recently, relating to an increased risk for ischemic stroke development within one year after their diagnosis.

This study analyzed the risk factor of other accompanying diseases—including anemia—for ischemic stroke development. As a hematological manifestation that can be caused by various acute and chronic conditions, patients with anemia showed a higher ratio of accompanying diseases posing as risk factors for ischemic stroke than those without. This tendency can be noted in previous results [4,20]. Iron deficiency anemia, the most common anemia world-wide, has been suggested to be associated to ischemic stroke in previous literatures [13,23]. Moreover, anemia can be a marker that reflects the overall health status associated with chronic diseases. Anemia is often associated with several underlying disorders, related or not to the anemia itself, requiring consideration for thorough investigation [10,12,24,25]. As non-infectious and infectious systemic inflammatory conditions caused by co-morbid disease can result in anemia, as well as a trigger for stroke vulnerability, anemia and ischemic stroke share a significant proportion of patients [4,20].

Chronic kidney disease is a common disease that can be caused by anemia as renal functions worsen. Out of the patients with chronic kidney disease, the reported prevalence of anemia was about 15%, which is over twice that of those without renal disease [26]. Diabetic nephropathy is known to have a prevalence of 26%–58% in diabetic patients, and the chance is even higher if accompanied by anemia [24]. Diabetic patients without reduced renal functions are known to have an approximately doubled risk of developing anemia compared to healthy adults [27]. Additionally, the prevalence of anemia is reportedly higher for people with poor blood pressure regulation [25], which might have caused the different ratios of accompanying disease between anemia and control groups in this study. Anemia is frequently associated with atrial fibrillation and is known to increase the risk of developing new atrial fibrillation [28]. When we analyzed the effect of anemia with known conventional risk factors-such as hypertension, diabetes and atrial fibrillation-on the development of ischemic stroke, the risk ratio was comparable with the previous studies performed only with conventional risk factors [29,30].

In this study, the risk for ischemic stroke development was relatively higher in patients <50 years old than those ≥50 years old. However, generalizing the risk of ischemic stroke development as higher for the younger population is not warranted because the risks associated with hypertension and diabetes also tended to be high. Anemia can also be a risk factor for ischemic stroke in younger patients like other comorbidities. Additionally, we found that anemia poses a greater risk for ischemic stroke in women than men. A previous study reported anemia as a risk factor for ischemic stroke regardless of sex [11], whereas another study reported it as only a risk factor in women similar to this study [20]. According to previous studies, the primary pathological mechanism of anemia implicated in ischemic stroke as a causative factor is impaired oxygen delivery due to the lack of hemoglobin. Lack of hemoglobin can reduce the oxygen delivered to both the brain and distal tissues, causing ischemic injuries [31]. This may explain why the risk for ischemic stroke is higher for women; the tolerance level of oxygen delivery capability may be lower in women when the hemoglobin level is reduced [32]. In pre-menopausal women, stroke incidence is smaller than in men due to estrogen’s protective effect [33]. The estrogen level and cerebral blood flow increase are known to be positively correlated [34]. Estrogen deficiency promotes structural and functional changes in blood vessels to increase the cardiovascular disease development risk [35]. Therefore, women without estrogen’s protection in the post-menopausal period may have a higher risk of stroke development in an anemic state.

In men, the ratio of smoking and alcohol consumption, which are another risk factors of ischemic stroke, is higher than that of women. However, the analysis of these risk factors could not be performed in this study. The prevalence of peripheral arterial occlusive disease (PAOD) is also higher in men than in women, and smoking is known to be the strongest risk factor for PAOD [36]. In our study, PAOD tends to be related to the risk of developing ischemic stroke in men, unlike women, possibly because it is related to smoking. It is possible that anemia did not show statistical significance on the risk of developing ischemic stroke in men due to the influence of risk factors not included in the analysis of this study.

Thrombus formation is another mechanism known to cause ischemic stroke. Anemia causes hyperdynamic circulation, which upregulates the molecular adhesion expression on vascular endothelial cells [37]. Consequently, the inflammatory response is triggered to form a thrombus, similar to how atherosclerosis is formed [38]. In iron-deficiency anemia, erythropoietin—secreted to increase the number of red blood cells—secretion increases to stimulate platelet formation, inducing thrombocytosis to create a thrombus [39].

This study identified an approximately 1.5-fold higher mortality risk for patients who developed ischemic stroke one year after their diagnosis of anemia in a follow-up observation, which is similar to or lower than the result from a previous meta-analysis [4,17]. However, direct comparison is inappropriate due to the difference in the selection method for research subjects. Previous study analyzed the mortality risk targeting patients with anemia, who already had a diagnosis of ischemic stroke. Therefore, the ratio of anemia was likely to be correlated with the prevalence of general population, but it was not possible to clarify since when anemia had started. In this study, there is a distinct difference in the characteristics of the studied subjects. In our study, mortality risk was analyzed by long-term follow-up observation of patients who developed ischemic stroke out of the extracted newly diagnosed with anemia from the total population. This study found that only atrial fibrillation and anemia—out of all accompanying diseases considered risk factors for ischemic stroke—were associated with mortality risk. The mortality risk is known to be higher for cerebral infarct patients with atrial fibrillation [40]. Multiple potential mechanisms have been suggested for how anemia causes an increase in mortality risk after ischemic stroke. Anemia is more common among older adults and likelier to be accompanied by other diseases [4]. Additionally, anemia can impair cerebrovascular autoregulation to cause cerebral perfusion fluctuations, exacerbating ischemia [4]. Further, inflammatory markers such as c-reactive protein, tumor necrosis factor-alpha and interleukins are increased in patients with anemia and associated with an adverse prognosis after stroke [17].

This study has several limitations. First, accurate hemoglobin levels were not known because subjects were extracted from claims data. The degree of risk of ischemic stroke development depends on the severity of anemia [20], which was not reflected in our analysis. The severity level of ischemic stroke can play a significant role in mortality after ischemic stroke and anemia is known to be associated with mortality risk for patients with an ischemic stroke with minor symptom, relatively [17]. We were unable to retrieve clinical data reflecting the severity level of ischemic stroke limited by the characteristics of claims data, so analysis was not conducted in relation to this. Due to the nature of the claim data, the possibility of biased result cannot be ruled out, since we could not analyze the traditional risk factors for ischemic stroke such as such as smoking, alcohol and obesity. Patients taking antiplatelet drugs or anticoagulants may have a low risk of developing ischemic stroke, but these factors were not analyzed. Even in patients with anemia and atrial fibrillation, non-vitamin K antagonist is known to lower the risk of developing ischemic stroke and major bleeding than warfarin [28]. However, we tried to minimize the number of study subjects taking antiplatelet or anticoagulant drugs, excluding patients who had previously had ischemic stroke.

It is known that the prevalence of anemia is associated with the socioeconomic status, especially in adolescent girls [41]. There may be differences in socioeconomic status between the anemia group and the control group in our study subjects, but this was not reflected in the analysis of the risk of ischemic stroke and death. In developing countries, anemia is mainly caused by nutritional deficiencies, but in high-income countries, dietary habits or other pathologic conditions are more common [42]. Considering the subjects of our study are adults from high-income country, the association between anemia and socioeconomic factors may not be significant.

Lastly, the risk evaluation of anemia alone by excluding other accompanying diseases’ influence on ischemic stroke development was a challenge because we failed to proportionate the rates of accompanying diseases when selecting research subjects. Still, our study is advantageous because we used an extensive database that represents the entire population of a nation to select subjects and analyzed a large sample, thus increasing the statistical results’ reliability. Additionally, we analyzed anemia’s influence after the development of ischemic stroke by confirming whether ischemic stroke developed in a short period of one to two years compared to previous studies. In longer-term investigations, the influence of other chronic diseases may be greater than anemia on the development of ischemic stroke, so short-term confirmation of the degree of risk may have reflected the influence by anemia more accurately.

## 5. Conclusions

The present study identified increased risk factors for ischemic stroke within two years after a new diagnosis of anemia. Thus, anemia should be investigated in patients who possess other risk factors of ischemic stroke. Furthermore, we highly encourage to carefully investigate and effectively manage anemia after ischemic stroke because of the associated mortality risk.

## Figures and Tables

**Table 1:** Demographic characteristics of subjects with anemia and without anemia extracted from sample cohort.

Variables	Anemia (*n* = 58,699)		Control (*n* = 117,398)		*p*-Value
	*n*	(%)	*n*	(%)	
Sex					0.4593
Male	18,907	(32.2%)	37,609	(32.0%)	
Female	39,792	(67.8%)	79,789	(68.0%)	
Age (years)					0.9722
18–29	4754	(8.1%)	9508	(8.1%)	
30–39	8179	(13.9%)	16,358	(13.9%)	
40–49	11,416	(19.4%)	22,832	(19.4%)	
50–59	8292	(14.1%)	16,584	(14.1%)	
60–69	9018	(15.4%)	18,212	(15.5%)	
≥70	17,040	(29.0%)	33,904	(28.9%)	
Comorbidities					
Hypertension	18,479	(31.5%)	26,878	(22.9%)	<0.0001
Diabetes mellitus	10,171	(17.3%)	9550	(8.1%)	<0.0001
Dyslipidemia	5973	(10.2%)	8684	(7.4%)	<0.0001
Chronic kidney disease	1289	(2.2%)	121	(0.1%)	<0.0001
Atrial fibrillation	930	(1.6%)	725	(0.6%)	<0.0001
PAOD	2443	(4.2%)	3271	(2.8%)	<0.0001
Previous ischemic stroke	2510	(4.3%)	1763	(1.5%)	<0.0001

PAOD: peripheral arterial occlusive disease.

**Table 2:** Analysis of the risk of ischemic stroke in patients diagnosed with anemia.

	Stroke within 1 Year			*p*-Value	Stroke within 2 Years			*p*-Value
	Adjusted OR	95% CI			Adjusted OR	95% CI		
		Lower	Upper			Lower	Upper	
Anemia	1.602	1.363	1.883	<0.0001	1.347	1.191	1.523	<0.0001
Hypertension	3.236	2.714	3.859	<0.0001	3.206	2.807	3.663	<0.0001
Diabetes mellitus	2.176	1.809	2.617	<0.0001	2.307	2.006	2.653	<0.0001
Dyslipidemia	0.789	0.614	1.013	0.064	0.797	0.659	0.963	0.019
Chronic kidney disease	0.881	0.480	1.617	0.683	1.034	0.663	1.611	0.883
Atrial fibrillation	4.161	2.929	5.910	<0.0001	3.711	2.787	4.940	<0.0001
PAOD	1.279	0.933	1.755	0.126	1.272	0.999	1.620	0.051

OR: odds ratio, CI: confidence intervals, PAOD: peripheral arterial occlusive disease.

**Table 3:** Adjusted odds ratio of anemia for the risk of ischemic stroke within 2 years in patients: subgroup analysis.

	20–49 Years Old (*n* = 72,912)				Over 50 Years Old (*n* = 98,912)				Men (*n* = 54,332)				Women (*n* = 117,492)			
	Adjusted OR	95% CI		*p*-Value **	Adjusted OR	95% CI		*p*-Value **	Adjusted OR	95% CI		*p*-Value **	Adjusted OR	95% CI		*p*-Value **
		Lower	Upper			Lower	Upper			Lower	Upper			Lower	Upper	
Anemia	2.404	1.232	4.689	0.010	1.414	1.247	1.605	<0.0001	1.243	1.037	1.489	0.0185	1.485	1.254	1.759	<0.0001
Hypertension	3.359	1.428	7.901	0.006	1.860	1.632	2.119	<0.0001	2.065	1.714	2.487	<0.0001	4.567	3.782	5.514	<0.0001
Diabetes mellitus	5.059	2.108	12.142	0.000	1.881	1.639	2.158	<0.0001	2.130	1.751	2.591	<0.0001	2.206	1.812	2.686	<0.0001
Dyslipidemia	2.003	0.751	5.345	0.165	0.702	0.579	0.850	0.0003	0.925	0.709	1.208	0.5684	0.692	0.529	0.905	0.0073
Chronic kidney disease	2.930	0.792	10.845	0.107	0.945	0.586	1.522	0.8154	0.869	0.493	1.530	0.6263	1.208	0.589	2.475	0.6059
Atrial fibrillation	*	*	*	*	3.160	2.377	4.201	<0.0001	2.295	1.451	3.628	0.0004	5.201	3.598	7.517	<0.0001
PAOD	*	*	*	*	1.140	0.896	1.450	0.2861	1.670	1.208	2.309	0.0019	0.945	0.657	1.360	0.7610

* Statistical analysis was not possible due to the small number of subjects with disease. ** Bonferroni correction for multiple testing was applied to adjust significant *p*-value at 0.0125. OR: odds ratio, CI: confidence intervals, PAOD: peripheral arterial occlusive disease.

**Table 4:** Analysis of the risk of death in patients diagnosed with ischemic stroke.

	Total Patients (*n* = 623)				Patients over 50 Years Old (*n* = 598)			
	HR	95% CI		*p*-Value	HR	95% CI		*p*-Value
		Lower	Upper			Lower	Upper	
Anemia	1.509	1.197	1.902	0.0005	1.536	1.216	1.940	0.0003
Hypertension	1.186	0.936	1.504	0.1583	1.121	0.881	1.427	0.3524
Diabetes mellitus	0.965	0.752	1.237	0.7777	0.949	0.738	1.221	0.6856
Dyslipidemia	1.079	0.744	1.565	0.6892	1.008	0.682	1.490	0.9663
Chronic kidney disease	0.726	0.296	1.783	0.4853	1.123	0.455	2.772	0.8016
Atrial fibrillation	3.324	2.251	4.909	<0.0001	3.256	2.204	4.808	<0.0001
PAOD	0.711	0.422	1.199	0.2007	0.702	0.416	1.185	0.1856

HR: hazard ratio, CI: confidence intervals, PAOD: peripheral arterial occlusive disease.

## Data Availability

The data presented in the study are available from the corresponding author on reasonable request.
